# Temporal clustering of prey in wildlife passages provides no evidence of a prey-trap

**DOI:** 10.1038/s41598-020-67340-8

**Published:** 2020-07-13

**Authors:** April Robin Martinig, Mahnoor Riaz, Colleen Cassady St. Clair

**Affiliations:** 1 Department of Biology, Concordia University, 1455 de Maisonneuve Boulevard W, Montreal, QC H3G 1M8 Canada; 2 Department of Biological Sciences, University of Alberta, Edmonton, AB T6G 2E9 Canada

**Keywords:** Ecology, Behavioural ecology, Conservation biology, Urban ecology

## Abstract

Wildlife passages are structures built across roads to facilitate wildlife movement and prevent wildlife collisions with vehicles. The efficacy of these structures could be reduced if they funnel prey into confined spaces at predictable locations that are exploited by predators. We tested the so-called prey-trap hypothesis using remote cameras in 17 wildlife passages in Quebec, Canada from 2012 to 2015 by measuring the temporal occurrence of nine small and medium-sized mammal taxa (< 30 kg) that we classified as predators and prey. We predicted that the occurrence of a prey-trap would be evidenced by greater frequencies and shorter latencies of sequences in which predators followed prey, relative to prey–prey sequences. Our results did not support the prey-trap hypothesis; observed prey–predator sequences showed no difference or were less frequent than expected, even when prey were unusually abundant or rare or at sites with higher proportions of predators. Prey–predator latencies were also 1.7 times longer than prey–prey sequences. These results reveal temporal clustering of prey that may dilute predation risk inside wildlife passages. We encourage continued use of wildlife passages as mitigation tools.

## Introduction

The negative ecological effects of road networks worldwide are extensive and well-documented^1–3^. For many species of wildlife, these negative effects include direct mortality from wildlife-vehicle collisions^4, 5^ and barriers to animal movement^2, 6, 7^. By reducing the functional connectivity of landscapes, roads can restrict dispersal and gene flow to subdivide populations, ultimately reducing genetic diversity^2, 3^. Barrier effects may be especially prevalent for small animals with limited mobility^6, 7^ and high sensitivity to road surfaces and traffic^8, 9^.

The barrier effects of roads can be mitigated with wildlife passages: structures designed or installed to facilitate animal movement over or under a road^10, 11^. Although road mitigation for large mammals has attracted more public attention, wildlife passages are also effective for small species of vertebrates, including small mammals^12–14^. However, these structures have the potential to alter relationships between prey and predators^15^, including by funnelling animals into confined spaces that might be exploited by predators to trap prey^16, 17^. The prey-trap hypothesis predicts that predators learn to exploit wildlife passages if the structures concentrate animal movement in predictable locations, thereby increasing prey detection and capture^12, 17–19^. Among mammals, both prey and predators emphasize olfactory and visual cues to detect the recent presence of other animals^20^ and the presence of predator feces or urine can induce avoidance behaviour in prey or attraction by predators^21, 22^.

Only a handful of studies have empirically tested the prey-trap hypothesis in the context of wildlife passages^17, 19, 23, 24^, but these offer insufficient evidence to determine the generality of a prey-trap effect^18, 25^. In the context of wildlife passages, a prey-trap could occur via several mechanisms. For example, high local abundance of prey could increase dispersal rates through wildlife passages to concentrate prey and attract predators. Conversely, low prey abundance might increase the value of targeting prey at predictable locations. Additionally, if wildlife passages are used more often by dispersing young of the year, targeting those locations might increase predator access to naïve individuals that are easier to catch^26, 27^, or naïve individuals might be more likely to use the kinds of structures that are favoured by predators^28^. Small mammalian predators (< 30 kg) appear to prefer particular wildlife passages, such as those that offer a dry substrate on concrete ledges^29^ or limited visibility^13, 29^ and those preferences may lead to exploitation by predators.

The objective of this study was to test the prey-trap hypothesis in a community of small mammals occupying a forested environment dissected by a four-lane road in the Laurentian Wildlife Reserve, Quebec, Canada. We did so by documenting individuals of nine taxa with remote cameras in 17 wildlife passages and determining the sequences of prey and predators. If predators targeted wildlife passages as prey-traps, we predicted that they would follow prey more often than predicted from the proportion of prey and predators. In addition to this general prediction, we sought evidence of a prey-trap at the times and locations where predators might be more likely to target prey, such as when they were either abundant or rare, when naïve animals were dispersing, or in the kinds of structures favoured by predators. Finally, we measured the latency between visits by successive individuals, predicting that attempts by predators to target prey would result in shorter prey–predator intervals relative to prey–prey or predator–predator sequences.

## Materials and methods

### Study site

We conducted our study between May 2012 and August 2015 along 65 km of Highway 175 in the Laurentides Wildlife Reserve in Quebec, Canada (47º34′ N, 71º12′ W) in boreal forest habitat. In response to increased traffic volume and highway twinning (see Martinig and Bélanger-Smith^29^ for details), continuous exclusion fencing was installed along 67 km of the highway (mesh size: 30 cm × 18 cm) along with wildlife passages accessible from within the exclusion fencing for both large and small mammals, with additional fencing (mesh size: 6 cm^2^) installed adjacent to all wildlife passages for up to 200 m^30^. We monitored 17 wildlife passages, each assigned a name that corresponds to the nearest kilometre marker, modified from existing drainage structures that targeted small and medium-sized mammals (< 30 kg). Wildlife passages were grouped into three types: pipe culverts (*n* = 6, average openness 0.009 ± 0.00005 SE), box culverts with a dry concrete ledge (*n* = 7, average openness 0.10 ± 0.001 SE), and box culverts with a wooden ledge (*n* = 4, average openness 0.25 ± 0.004 SE) (see Martinig and Bélanger-Smith^29^ for details of wildlife passage construction). Openness was calculated as width × height/length^31^. Elevation at the sites of wildlife passages ranged from 476 to 820 m. Monitored wildlife passages were an average of 3.8 km ± 0.82 SE apart with the closest two wildlife passages located 323 m apart. This spacing is assumed to minimize spatial autocorrelation to support independence of observations between sampling sites because home range sizes were under 1 km^2^ for all mammals considered here except American mink (*Neovison vison*; hereafter mink; 1.5–16 km^2^)^32^.

### Study species

We included nine taxonomic groups in this study, which we identified as species (below) or grouped in taxonomic levels that could be identified as weasels (*Mustela* spp*.* included *Mustela frenata* and *Mustela erminea*), and micromammals (shrews (Family Soricidae), mice (Family Cricetidae), moles (Family Talpidae), and voles (Family Cricetidae)). The species we classified as predators included mink, weasels, and red foxes (*Vulpes vulpes*). We described North American red squirrels (*Tamiasciurus hudsonicus*, hereafter red squirrel) as prey instead of predators because they subsist mainly on conifer seeds^33^, despite including smaller mammals and birds in their diet^33–38^. However, we conducted separate analyses that excluded red squirrels from our analyses or treated them as predators to demonstrate that these classifications did not change our conclusions about the existence of a prey-trap (Supporting Information, Tables S1 and S2). Mink are typically sit-and-wait predators whereas red foxes and weasels actively chase and hunt prey^32^. We did not observe any larger predators (e.g. wolves (*Canis lupus*), coyotes (*Canis latrans*), or Canada lynx (*Lynx canadensis*)) using the wildlife passages. We classified herbivores as prey and included Eastern chipmunks (*Tamia striatus*), marmots (*Marmota monax*), common muskrats (*Ondatra zibethicus*), snowshoe hares (*Lepus americanus*), and micromammals^32^.

### Data collection

Wildlife passages were equipped with remote cameras (Reconyx HC600 Hyperfire H.D. Covert IR, Holmen, WI, USA) to detect animals through a combination of heat and movement. We placed one camera at each entrance facing inwards at the openings of wildlife passages^29^. Following activation, five photographs were taken at up to two frames per second for each triggering event. Because snow cover and flooding caused some camera failures, we limited our observational period to times with no disturbances (June to October in all years except 2015 when the study ended in August). Although motion-activated cameras can be biased toward species that are slow-moving^39^, large^39, 40^, or motile^41^, we assumed that these differences did not apply to our community of mammals.

For each series of five photos (i.e., a triggering event), we recorded location, date, temperature, time, and species. We estimated size of individuals using a reference block placed inside each wildlife passage and in the cameras’ field of view to increase our ability to differentiate between individuals of the same species. We assumed temporal independence for observations of the same species that were greater than 10 min apart unless successive individuals were of the same species and size, in which case we extended this limit to 20 min (after Martinig and Bélanger-Smith^29^). We included in our analyses only the first picture for these sequences and excluded observations of individuals we could not attribute to taxa owing to poor lighting or camera angles (2.6% of observations). This study was approved by and was carried out in accordance with relevant guidelines and regulations as set out by the Quebec Ministry of Transportation (approval number: R709.1) and Concordia University (animal care ethics protocol number: 30001331).

### Data analysis

Using the trophic positions (prey and predator) and independent observations (camera detections) described above, we identified pairwise sequences for all successive observations at each wildlife passage for each monitoring season wherein *n* observations resulted in *n* − 1 pairwise sequences. We attributed these sequences to one of four possible combinations: prey followed by prey (prey–prey); prey followed by predator (prey–predator); predator followed by prey (predator–prey); and predator followed by predator (predator–predator). All analyses were done in R, version 3.6.2^42^. Data are reported as means ± one SE.

#### Sequence proportions

We tested our first prediction, that the proportion of prey–predator sequences was higher than expected, by calculating the proportion of the sample comprised by prey and predators and applying the binomial distribution to generate the randomly-expected probabilities for each of the four sequences. For each wildlife passage, we recorded the number of prey (*x*) and predators (*y*), sample size (*n*), sequences (*n − *1), and number of prey–predator sequences (*k*_*a*_). Stemming from the prey-trap hypothesis that predators detect prey use of wildlife passages, we predicted that the number of prey–predator sequences would be significantly higher than the number predicted by the binomial distribution, given the proportion of prey and predators in each sampling period.

We calculated the null probability (*p*_*e*_) by wildlife passage for the prey–predator sequence using the equation:

$$p_{e} \left( {y|x} \right) \, = \frac{x}{n} \cdot \frac{y}{n}$$

We calculated the expected number of prey–predator sequences (*k*_*e*_) given *p*_*e*_ using the following equation:

$$k_{e} = \, (n - {1}) \cdot p_{e}$$

We conducted a two-tailed binomial test using the *binom.test* function in the baseline ‘stats’ package in R, version 3.6.2^42^, to obtain the observed probability (*p*_*a*_) of obtaining ≥ *k*_*a*_ prey–predator sequences in *n − *1 sequences for each wildlife passage. The binomial distribution uses the following equation for 0 < *p*_*e*_ < 1 and *p*_*a*_ > 0:

$$p_{a} = \left( {\begin{array}{*{20}c} n \\ k \\ \end{array} } \right)p_{e}^{ka} ({1} - p_{e} )^{n - ka}$$

where *k*_*a*_ is 0, 1, 2,…, n:

$$\left( {\begin{array}{*{20}c} n \\ k \\ \end{array} } \right) = \frac{n!}{{k!\left( {n - k} \right)!}}$$

In addition to calculating the observed probability (*p*_*a*_), the *binom.test* function provided the 95 percent confidence intervals (95% CIs) around *p*_*a*_ and the *p*-value. A significant effect was inferred if the observed probability ± 95% CIs did not overlap with its corresponding null probability. We included all sequences without temporal censoring here for two reasons, 1) the infrequency of use by predators and prey is biologically meaningful in the context of a prey-trap, and 2) scent trails left by prey typically last between 24 h and 1 week^43, 44^. We also explored the effect of temporal censoring on our results to acknowledge scent depletion (see below).

#### Biologically relevant metrics: restricted analyses

We tested our second prediction, that the proportion of prey–predator sequences would increase under certain ecological conditions, by restricting our analyses to the associated periods or structures. First, we limited our analysis to the peak and trough of the regional population sizes for small mammals during our study, which occurred in an area that is known to exhibit population cycles^45^. Both prey and predators exhibited their highest abundances in 2012 and their lowest abundances in 2015. We conducted a second restricted analysis for the dispersal period in August, when juveniles are more prevalent for many species of small mammals^32^. Last, we analyzed a subset of wildlife passage types that experienced high predator use in a simultaneous study^29^; those with limited visibility (openness < 0.089) and box culverts with a dry concrete ledge.

#### Latency between sequences

We tested our third prediction, that the latency differed among the four possible sequences (prey–prey, prey–predator, predator–prey, and predator–predator), by measuring the time between the first and second detection. Again, we classified individuals as prey or predator and ordered the observations by ascending time for each wildlife passage. We then calculated the geometric mean time between detections starting from the first observation in each June to October period. We determined the geometric mean latency for all four possible sequences of prey and predators in each wildlife passage (wildlife passage-specific latency) and then averaged across all wildlife passages for an overall latency. We used the geometric mean instead of the arithmetic mean to reduce the weight of extreme values.

We fit the latency models (all sequences or restricted to sequences ≤ 24 h of each other) using a negative binomial regression with the “glmmTMB” package, version 1.0.1^46^, because the variable’s variance was larger than its mean. We included time between observations (in days for the global model and hours for the restricted model) as the response variable and sequence type, wildlife passage type, openness, year, and month as fixed effects and wildlife passage identity as a random effect. We mean-centered and standardized openness to one standard deviation^47^. We estimated pairwise comparisons using the “lsmeans” package, version 2.30–0^48^.

## Results

We detected 11,278 mammals that we considered independent observations at the 17 wildlife passages between June and October of 2012 to 2015. During this time, we did not observe a single predation attempt or event on our remote cameras. We classified 88% of the detections as prey and 12% as predators. In wildlife passages, the number of prey detections ranged from 19–1521 (31–97% of detections among wildlife passages) and the number of predator detections ranged from 14 to 254 (3–69% of detections among wildlife passages; Table 1). The most prevalent taxa observed were micromammals (58%, *n* = 6,576), red squirrels (15%, *n* = 1632), marmots (11%, *n* = 1,215), weasels (9%, *n* = 960), and mink (4%, *n* = 409). The remaining four taxa encompassed 4% of observations (snowshoe hares: *n* = 217, Eastern chipmunks: *n* = 141, common muskrats: *n* = 111, and red foxes: *n* = 17).

**Table 1:** Information on passage type and mammal sequence data for 17 wildlife passages located along Highway 175 in the Laurentian Wildlife Reserve, Quebec, Canada from 2012 to 2015. Number of detections on remote cameras are provided for prey, predators, and pairwise sequences of successive detections. The number of prey–predators and prey–prey sequences (*k*) are tallied for observed sequences (*k*_*a*_) and expected sequences (*k*_*e*_) based on the binomial distribution of observed (*p*_*a*_) and expected (*p*_*e*_) proportions, with corresponding p-values and 95% confidence intervals (CI) from a two-tailed binomial test. Asterisk denotes significance at α = 0.05.

Passage number	Number of sequences		Prey		Predators		Prey–predators sequences (*k*_*a*_ (*k*_*e*_))		Proportions (*p*_*a*_ (*p*_*e*_))		*P* (95% CI)		Prey–prey sequences (*k*_*a*_ (*k*_*e*_))		Proportions (*p*_*a*_ (*p*_*e*_))	***P (95% CI)***	
80	93		81		13		12 (11)		0.13 (0.12)		0.75 (0.07, 0.21)		69 (70)		0.74 (0.74)	1.00 (0.64, 0.83)	
81	1541		1,408		134		103 (122)		0.07 (0.08)		0.07 (0.05, 0.08)		1,305 (1,285)		0.85 (0.83)	0.17 (0.83, 0.86)	
83	316		305		12		5 (12)		0.02 (0.04)		0.05* (0.01, 0.04)		300 (293)		0.95 (0.93)	0.13 (0.92, 0.97)	
84	721		659		63		44 (57)		0.06 (0.08)		0.07 (0.04, 0.08)		615 (601)		0.85 (0.83)	0.16 (0.83, 0.88)	
89	866		735		135		58 (114)		0.07 (0.13)		< 0.001* (0.05, 0.09)		674 (617)		0.78 (0.71)	< 0.001* (0.75, 0.81)	
89.5	167		149		19		14 (17)		0.08 (0.10)		0.61 (0.05, 0.14)		135 (131)		0.81 (0.79)	0.57 (0.74, 0.87)	
96	225		169		57		21 (42)		0.09 (0.19)		< 0.001* (0.06, 0.14)		148 (126)		0.66 (0.56)	< 0.01* (0.59, 0.72)	
99	407		367		41		19 (37)		0.05 (0.09)		< 0.01* (0.03, 0.07)		348 (329)		0.86 (0.81)	0.02* (0.82, 0.89)	
104	56		19		38		12 (12)		0.21 (0.22)		1.00 (0.12, 0.34)		7 (6)		0.13 (0.11)	0.67 (0.05, 0.24)	
107	1691		1519		173		88 (155)		0.05 (0.09)		< 0.001* (0.04, 0.06)		1,431 (1,363)		0.85 (0.81)	< 0.001* (0.83, 0.86)	
110	58		48		14		9 (10)		0.16 (0.18)		0.73 (0.07, 0.27)		36 (34)		0.62 (0.58)	0.60 (0.48, 0.75)	
122	190		127		64		37 (42)		0.19 (0.22)		0.38 (0.14, 0.26)		90 (84)		0.47 (0.44)	0.38 (0.40, 0.55)	
124	1581		1,425		157		115 (141)		0.07 (0.09)		0.02* (0.06, 0.09)		1,310 (1,283)		0.83 (0.81)	0.08 (0.81, 0.85)	
125	880		725		156		85 (128)		0.10 (0.15)		< 0.001* (0.08, 0.12)		640 (596)		0.73 (0.68)	< 0.01* (0.70, 0.76)	
133	1,375		1,122		254		135 (207)		0.10 (0.15)		< 0.001* (0.08, 0.12)		987 (914)		0.72 (0.67)	< 0.001* (0.69, 0.74)	
142	119		95		25		11 (20)		0.09 (0.17)		0.03* (0.05, 0.16)		84 (75)		0.71 (0.63)	0.09 (0.62, 0.79)	
144	975		945		31		16 (30)		0.02 (0.03)		< 0.01* (0.01, 0.03)		929 (914)		0.95 (0.94)	0.05* (0.94, 0.97)	
Sum	11,277		9,892		1,386		784 (1,216)		0.07 (0.11)		< 0.001* (0.06, 0.07)		9,108 (8,676)		0.81 (0.77)	< 0.001* (0.80, 0.82)	

**Figure 1:**
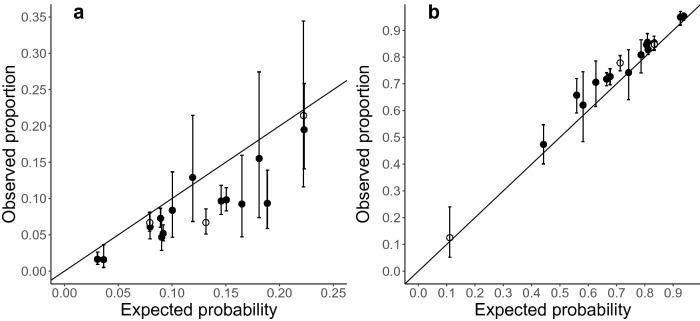
Observed proportion (*p*_*a*_) of (**a**) prey–predator and (**b**) prey–prey sequences with 95% confidence intervals obtained from binomial tests compared to corresponding expected (null) proportions (*p*_*e*_) of (**a**) prey–predator and (**b)** prey–prey sequences for 17 wildlife passages from 2012 to 2015. Wildlife passages where predator–predator sequences were most common in white. Line with slope = 1 plotted.

As expected from the greater proportion of prey in our observations, prey–prey sequences were observed most often overall and in 16 of the 17 wildlife passages (Fig. S1). However, there was considerable variation in the proportion of sequence types among wildlife passages and predator–predator sequences exhibited their highest percentages (50%) in the wildlife passage in which predators were more abundant than prey (Table 1; Fig. 1; Fig. S1). Prey–prey sequences ranged from 13 to 95% among wildlife passages (Table 1; Fig. S1).

### Proportion of prey–predator sequences

If predators track prey in wildlife passages, we predicted that prey–predator sequences would occur more often than expected from the proportion of prey and predators. Our results did not support this prediction, instead exhibiting a significant departure in the other direction. When all 17 wildlife passages were combined, the observed proportion of a prey–predator sequence was lower than expected (Table 1; *p*_*a*_ = 0.07, 95% CI (0.06, 0.07), *p*_*e*_ = 0.11, *p* < 0.001). Among the 17 wildlife passages we monitored, ten (59%) exhibited significantly lower proportions of prey–predator sequences than expected, while the other seven (41%) showed no difference between observed and expected proportions of prey–predator sequences (Table 1; Fig. 1a). We present only the frequencies for prey–predator sequences in the main results because the frequencies of predator–prey sequences are ± 1 value of the prey–predator sequences within each wildlife passage by year (Table S3; Fig. S3).

### Proportion of prey–prey sequences

In contrast to the lack of support for a prey-trap hypothesis, we found evidence in seven (41%) of the wildlife passages that prey were more likely to follow prey than would be expected by their numerical frequency alone (Table 1; Fig. 1b). When all 17 wildlife passages were combined, the observed proportion of a prey–prey sequence was higher than expected (Table 1; *p*_*a*_ = 0.81, 95% CI (0.80, 0.82), *p*_*e*_ = 0.77, *p* < 0.001). The remaining 10 wildlife passages (59%) showed no difference between observed and expected proportions of prey–prey sequences (Table 1).

### Biologically relevant metrics: restricted analyses

Similar to our analysis with the combined dataset, our second test was unsupportive of the prey-trap hypothesis when analyses were restricted to ecological conditions that might be expected to increase the benefits of a prey-trap to predators. When observations were restricted to 2012, when prey abundance was highest, or to 2015, the year with the lowest prey abundance, all wildlife passages showed either no difference or had lower observed proportions of prey–predator sequences than expected (Tables S4 and S5; Fig. S2). Results from observations limited to August were similar (Table S6; Fig. S2). Restricting the analyses to the kinds of wildlife passages with limited visibility or concrete ledges that are favoured by predators also failed to support the prey-trap hypothesis (Fig. S3). In all cases, observed proportions of prey–predator sequences did not differ from expected or were lower than expected (Table 1; Fig. S3).

### Latency between sequences

**Figure 2:**
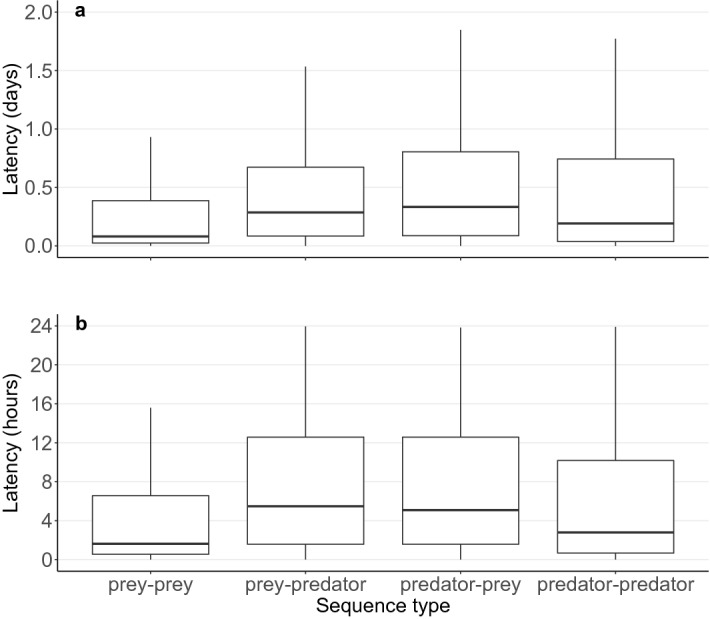
Box and whisker plots showing the latency for all sequence types for 17 wildlife passages from 2012 to 2015. (**a**) Latency for all sequences (days, *n* = 11,278) and (**b**) only sequences ≤ 24 h apart (hours, *n* = 9,650). Prey–predator latencies were longer than prey–prey latencies. Outliers were excluded to ease visualization.

Results for our third prediction about the latency between detections of different sequence types also failed to support the prey-trap hypothesis. The geometric mean latency across all wildlife passages was 20 h ± 9.6 SE for prey–prey sequences, 32 h ± 9.0 SE for prey–predator sequences, 30 h ± 7.1 SE for predator–prey sequences, and 18 h ± 5.6 SE for predator–predator sequences. Latency was significantly different between sequence types and wildlife passage types (Table 2; Fig. 2), with prey–prey sequences having the shortest latency and predator–prey sequences having the longest (Fig. 2). Latencies in August were shorter than in all other months and latencies increased with year (Table 2). There was no effect of openness on latency (Table 2). Results were consistent when considering only sequences ≤ 24 h apart (Table 2).

**Table 2:** Sources of variation in latency for all sequences (days, *n* = 11,278) and only sequences ≤ 24 h apart (hours, *n* = 9,650) for predator and prey occurrences in 17 wildlife passages from 2012 to 2015. We provide point estimates from univariate generalized linear mixed effects models for fixed effects (β) with 95% confidence intervals (CI) and the random effect (σ^2^; variance) ± one standard deviation (SD). Asterisk denotes significance at α = 0.05.

	All sequences (latency in days)		Sequences ≤ 24 h apart (latency in hours)	
Fixed effects^a^	β (95% CI)	*P*	β (95% CI)	***P***
Sequence^b^				
Prey–predator	0.72 (0.58, 0.85)	< 0.001*	0.40 (0.27, 0.54)	< 0.001*
Predator–prey	0.72 (0.58, 0.85)	< 0.001*	0.43 (0.29, 0.57)	< 0.001*
Predator–predator	0.52 (0.37, 0.67)	< 0.001*	0.07 (− 0.09, 0.23)	0.65
Comparison predator–prey to prey–predator	0.002 (− 0.17, 0.18)	1.00	0.02 (− 0.16, 0.21)	0.99
Comparison predator– predator to prey–predator	− 0.20 (0.01, 0.38)	0.03*	− 0.33 (− 0.53, − 0.13)	< 0.001*
Comparison predator– predator to predator–prey	− 0.20 (0.01, 0.38)	0.03*	− 0.35 (− 0.56, − 0.15)	< 0.001*
Wildlife passage type^b^				
Wooden ledge	1.44 (0.38, 2.49)	< 0.01*	0.27 (− 0.15, 0.69)	0.30
Pipe	1.76 (1.35, 2.62)	< 0.001*	0.47 (0.12, 0.83)	0.005*
Comparison wooden ledge to pipe	0.33 (− 2.53, 1.52)	0.80	0.21 (− 0.24, 0.65)	0.53
Openness	− 0.09 (− 0.54, 0.37)	0.71	− 0.02 (− 0.19, 0.15)	0.80
Year	0.40 (0.37, 0.43)	< 0.001*	0.20 (0.18, 0.23)	< 0.001*
Month	0.22 (0.15, 0.29)	< 0.001*	0.20 (0.15, 0.26)	< 0.001*
**Random effect**	**σ**^**2**^** ± SD**		**σ**^**2**^** ± SD**	
Wildlife passage identity	0.40 ± 0.63		0.05 ± 0.23	

^a^Reference categories for fixed effects were set to ‘prey–prey’ (sequence), ‘concrete ledge’ (wildlife passage type), ‘0’ (i.e., 2012 for year) and ‘August’ (month). Openness was mean centered and standardized to one standard deviation.

^b^Obtained from pairwise comparisons. Interpret as effect of the first variable relative to second.

## Discussion

Researchers have speculated that wildlife passages directing animals into a confined structure could result in predators using these structures to facilitate prey capture^12, 17, 39^. We tested this hypothesis using the temporal sequences of animals detected by remote cameras in 17 wildlife passages over a four-year period. If predators used wildlife passages as prey-traps, we predicted that prey detections would be followed by predator detections more often than expected given the frequency of prey and predators. We predicted this tendency would be even greater at the times and locations where ecological circumstances might increase the opportunity for a prey-trap to occur, such as when prey were especially abundant or rare, while naïve animals were dispersing, and in the kinds of crossing structures favoured by predators. We also expected that prey–predator sequences would exhibit shorter internals. None of these predictions were supported by our analyses, lending no support to this form of the prey-trap hypothesis. Instead, the proportion of sequences in which predators followed prey were either equal to or less than expected. Moreover, the proportion of sequences in which prey followed prey was higher than expected, while the latency between these detections was the lowest, together suggesting that temporal clustering by prey, whether incidental or intentional, might dilute their predation risk in wildlife structures.

The patterns we observed in this system appear to be driven by prey, which presumably used the wildlife passages in greater frequencies than predators because of their greater abundance in the surrounding landscape. Along with prey occurring at the highest frequency, prey–prey sequences showed the lowest latency periods, indicating closer proximity in time, and space, of prey to other prey. Although the species we studied do not travel in groups, this spatiotemporal grouping may have resulted from pulses of resource availability or foraging activity, which could produce temporal clusters of prey incidentally (e.g., in red squirrels; Boutin et al.^49^)). However, temporal clustering by prey could also occur if prey responded to cues from other prey^50, 51^ and exhibited greater gregariousness to dilute their risk of predation^52–54^. Such a mechanism would equate to the behavioural expectations of a selfish herd, as described by Hamilton^53^. This might also explain the greater latency of prey–predator sequences in our data. Our interpretation of such dilution is speculative, but consistent with other studies that found aversion to predators by prey^50, 55–57^.

An alternative to our conclusion that prey avoid a prey-trap (whether or not it was through temporal clustering by prey) is that predators track prey, but we failed to detect those efforts. A recent study inferred spatiotemporal avoidance from a significantly smaller sample size than seen here, while still not documenting interspecific interactions in the wildlife passages^23^. While we cannot rule out the possibility that the use of the wildlife passages here was still too infrequent for scent trails to be readily used in this study system this possibility is unlikely, especially given this earlier study. Although scent trails left by prey typically last 24 h^43^, even week-old scent trails can attract predators^44^. When we tested for an effect of scent age by restricting our analysis to sequences that occurred within one day, maximizing conditions for prey detection by predators, the observed frequencies of prey–predator sequences were no higher than for the whole sample. Similarly, there was no evidence of a prey-trap in the types of wildlife passages used most by predators, wildlife passages where they would have had more opportunity to detect scent trails from prey. Thus, diminished scent cues are an unlikely explanation for why we did not find support for the prey-trap hypothesis.

Our conclusions were similarly unchanged by restricting our analyses to ecological conditions that might be expected to increase the likelihood of a prey-trap. There were no differences in our results as a function of high (2012) or low (2015) prey abundance, the season (August) of high prey dispersal, or in the subset of wildlife passages (i.e., limited visibility or concrete ledges) that we know are favoured by predators on this landscape^29^. Each of these circumstances could have increased the benefit or ease of prey tracking by predators by increasing detection rates or learning opportunities by predators^26–29^.

A viable interpretation of our results is that low predator, relative to prey, abundance generally limits the likelihood of prey-traps at wildlife passages here and elsewhere^19, 24^. For a prey-trap to occur, predators would have to detect (i.e., visually or olfactorily) and encounter prey inside wildlife passages with sufficient frequency to develop search images (sensu^57–59^). Surprisingly, there have been no documented cases of predators killing prey in wildlife passages; there has only been one documented case of an (unsuccessful) predation attempt on an arboreal marsupial^25^ and the presence of a carcass near a wildlife passage^19^. Both encounter rates and capture success by predators may be too infrequent in wildlife passages for predators to learn to associate them with hunting opportunities (sensu^60, 61^), or otherwise influence their foraging behaviour^62^, particularly if small and medium-sized predators hunt differently from larger predators. By contrast, many species may use wildlife passages to forage in habitat in highway verges or the median, and predators may traverse wildlife passages with prey that they captured outside of them^63^.

## Conclusions

In summary, we found no support for the hypothesis that wildlife passages built to mitigate the barrier effects of roads could function as prey-traps for small and medium-sized mammals^17, 19^. If predators targeted prey in this way, we predicted that it would increase the interspersion of prey–predator sequences in wildlife passage use relative to the frequency predicted by prey and predator abundance alone. None of our results supported this prediction. Instead, the frequency of prey–prey sequences was higher than expected, with corresponding low latencies. We speculate that prey may dilute their predation risk through spatiotemporal clustering when using wildlife passages, either incidentally or adaptively. Our results are consistent with the few other studies that tested the prey-trap hypothesis in association with wildlife passages, suggesting such traps do not occur or are difficult to detect^19, 24^. Despite our lack of support for it, we suggest that the prey-trap hypothesis merits additional study in other systems, areas, and taxa. In the meantime, we support the conclusions by others that wildlife passages are effective tools for mitigating movement across roads for diverse taxa that include assemblages of prey and predators^10, 11^.

## Supplementary information

Supplementary file1
