# Supplementary material for: Temporal clustering of prey in wildlife passages provides no evidence of a prey-trap
Source: Sci Rep. 2020 Jul 13;10:11489. doi: 10.1038/s41598-020-67340-8 (PMC7359302; doi:10.1038/s41598-020-67340-8)
Supplement: Supplementary file 1 — Supplementary file1 [file 41598_2020_67340_MOESM1_ESM.docx]

**Supporting Information**

Martinig, A. R., M. Riaz, and C. C. St. Clair. 2020. Temporal clustering of prey in wildlife passages provides no evidence of a prey-trap. Scientific Reports.

**Table of contents**

Table S1. Sequence output excluding red squirrels. Page 1

Table S2. Sequence output treating red squirrels as predators. Page 2

Table S3. Predator–prey sequence output. Page 3

Table S4. Sequence output for 2012 only. Page 4

Table S5. Sequence output for 2015 only. Page 5

Table S6. Sequence output for August only. Page 6

Figure S1. Number of observations by wildlife passage. Page 7

Figure S2. Proportions for 2012 only, 2015 only, and August only. Page 8

Figure S3. Proportions by wildlife passage type and openness. Page 9

**Table S1.** Information on mammal sequence data (**excluding red squirrels**) for 17 wildlife passages located along Highway 175 in the Laurentian Wildlife Reserve, Quebec, Canada from 2012 to 2015. Number of detections on remote cameras are provided for prey, predators, and pairwise sequences of successive detections. The number of prey–predators sequences (*k*) are tallied for observed sequences (*k_a_*) and expected sequences (*k_e_*) based on the binomial distribution of observed (*p_a_*) and expected (*p_e_*) proportions, with corresponding p-values and 95 % confidence intervals (CI) from a two-tailed binomial test. Asterisk denotes significance at α = 0.05.

| **Passage** | **Number of sequences** | **Prey** | **Predators** | **Prey–predators sequences (*k_a_* (*k_e_*))** | **Proportions (*p_a_* (*p_e_*))** | ***P* (95 % CI)** |
| --- | --- | --- | --- | --- | --- | --- |
| 80 | 93 | 81 | 13 | 12 (11) | 0.13 (0.12) | 0.75 (0.07, 0.21) |
| 81 | 765 | 633 | 133 | 69 (110) | 0.09 (0.14) | < 0.001 * (0.07, 0.11) |
| 83 | 314 | 303 | 12 | 5 (12) | 0.02 (0.04) | 0.05 * (0.01, 0.04) |
| 84 | 527 | 465 | 63 | 42 (55) | 0.08 (0.11) | 0.06 (0.06, 0.11) |
| 89 | 416 | 283 | 134 | 39 (91) | 0.09 (0.22) | < 0.001 * (0.07, 0.13) |
| 89.5 | 165 | 147 | 19 | 14 (17) | 0.08 (0.10) | 0.61 (0.05, 0.14) |
| 96 | 225 | 169 | 57 | 21 (42) | 0.09 (0.19) | < 0.001 * (0.06, 0.14) |
| 99 | 407 | 367 | 41 | 19 (37) | 0.05 (0.09) | < 0.001 * (0.03, 0.07) |
| 104 | 56 | 19 | 38 | 12 (12) | 0.21 (0.22) | 1.00 (0.12, 0.34) |
| 107 | 1682 | 1510 | 173 | 88 (155) | 0.05 (0.09) | < 0.001 * (0.04, 0.06) |
| 110 | 57 | 44 | 14 | 9 (10) | 0.16 (0.18) | 0.73 (0.07, 0.28) |
| 122 | 179 | 116 | 64 | 34 (41) | 0.19 (0.23) | 0.25 (0.14, 0.26) |
| 124 | 1536 | 1380 | 157 | 114 (141) | 0.07 (0.09) | 0.02 * (0.06, 0.09) |
| 125 | 835 | 680 | 156 | 82 (127) | 0.10 (0.15) | < 0.001 * (0.08, 0.12) |
| 133 | 1319 | 1066 | 254 | 134 (205) | 0.10 (0.16) | < 0.001 * (0.09, 0.12) |
| 142 | 118 | 94 | 25 | 11 (20) | 0.09 (0.17) | 0.03 * (0.05, 0.16) |
| 144 | 929 | 899 | 31 | 15 (30) | 0.02 (0.03) | < 0.01 * (0.01, 0.03) |
| Sum | 9639 | 8256 | 1384 | 720 (1185) | 0.07 (0.12) | < 0.001 * (0.07, 0.08) |

**Table S2.** Information on passage type and mammal sequence data (**treating red squirrels as predators instead of prey**) from 2012 to 2015. See above (Table S1) for further details.

| **Passage** | **Number of sequences** | **Prey** | **Predators** | **Prey–predator sequences (*k_a_* (*k_e_*))** | **Proportions (*p_a_* (*p_e_*))** | ***P* (95 % CI)** |
| --- | --- | --- | --- | --- | --- | --- |
| 80 | 93 | 81 | 13 | 12 (11) | 0.13 (0.12) | 0.75 (0.07, 0.21) |
| 81 | 1541 | 633 | 909 | 228 (373) | 0.15 (0.24) | < 0.001 * (0.13, 0.17) |
| 83 | 316 | 304 | 13 | 7 (12) | 0.02 (0.04) | 0.15 (0.01, 0.05) |
| 84 | 721 | 465 | 257 | 90 (165) | 0.12 (0.23) | < 0.001 * (0.10, 0.15) |
| 89 | 866 | 285 | 582 | 111 (191) | 0.13 (0.22) | < 0.001 * (0.11, 0.15) |
| 89.5 | 167 | 147 | 21 | 15 (18) | 0.09 (0.11) | 0.53 (0.05, 0.14) |
| 96 | 225 | 169 | 57 | 21 (42) | 0.09 (0.19) | < 0.001 * (0.06, 0.14) |
| 99 | 407 | 367 | 41 | 19 (37) | 0.05 (0.09) | < 0.01 * (0.03, 0.07) |
| 104 | 56 | 19 | 38 | 12 (12) | 0.21 (0.22) | 1.00 (0.12, 0.34) |
| 107 | 1691 | 1510 | 182 | 93 (162) | 0.06 (0.10) | < 0.001 * (0.04, 0.07) |
| 110 | 58 | 44 | 15 | 10 (11) | 0.17 (0.19) | 0.87 (0.09, 0.29) |
| 122 | 190 | 116 | 75 | 39 (45) | 0.21 (0.24) | 0.31 (0.15, 0.27) |
| 124 | 1581 | 1380 | 202 | 140 (176) | 0.09 (0.11) | < 0.01 * (0.08, 0.10) |
| 125 | 880 | 680 | 201 | 101 (155) | 0.11 (0.18) | < 0.001 * (0.09, 0.14) |
| 133 | 1375 | 1066 | 310 | 159 (240) | 0.12 (0.17) | < 0.001 * (0.10, 0.13) |
| 142 | 119 | 94 | 26 | 12 (20) | 0.10 (0.17) | 0.05 * (0.05, 0.17) |
| 144 | 975 | 900 | 76 | 52 (70) | 0.05 (0.07) | 0.03 * (0.04, 0.07) |
| Sum | 11277 | 8260 | 3018 | 1121 (2210) | 0.10 (0.20) | < 0.001 * (0.09, 0.11) |

**Table S3.** Information on **predator–prey**^a^ sequence data from 2012 to 2015. See above (Table S1) for further details.

| **Passage** | **Number of sequences** | **Prey** | **Predators** | **Predator–prey sequences (*k_a_* (*k_e_*))** | **Proportions (*p_a_* (*p_e_*))** | ***P* (95 % CI)** |
| --- | --- | --- | --- | --- | --- | --- |
| 80 | 93 | 81 | 13 | 11 (11) | 0.12 (0.12) | 1.00 (0.06, 0.20) |
| 81 | 1541 | 1408 | 134 | 102 (122) | 0.07 (0.08) | 0.06 (0.05, 0.08) |
| 83 | 316 | 305 | 12 | 3 (12) | 0.01 (0.04) | < 0.01 * (0.00, 0.03) |
| 84 | 721 | 659 | 63 | 44 (57) | 0.06 (0.08) | 0.07 (0.04, 0.08) |
| 89 | 866 | 735 | 135 | 58 (114) | 0.07 (0.13) | < 0.001 * (0.05, 0.09) |
| 89.5 | 167 | 149 | 19 | 12 (17) | 0.07 (0.10) | 0.25 (0.04, 0.12) |
| 96 | 225 | 169 | 57 | 21 (42) | 0.09 (0.19) | < 0.001 * (0.06, 0.14) |
| 99 | 407 | 367 | 41 | 20 (37) | 0.05 (0.09) | < 0.01 * (0.03, 0.07) |
| 104 | 56 | 19 | 38 | 10 (12) | 0.18 (0.22) | 0.52 (0.09, 0.30) |
| 107 | 1691 | 1519 | 173 | 87 (155) | 0.05 (0.09) | < 0.001 * (0.04, 0.06) |
| 110 | 58 | 48 | 14 | 8 (11) | 0.14 (0.18) | 0.50 (0.06, 0.25) |
| 122 | 190 | 127 | 64 | 34 (42) | 0.18 (0.22) | 0.16 (0.13, 0.24) |
| 124 | 1581 | 1425 | 157 | 115 (141) | 0.07 (0.09) | 0.02 * (0.06, 0.09) |
| 125 | 880 | 725 | 156 | 84 (128) | 0.10 (0.15) | < 0.001 * (0.08, 0.12) |
| 133 | 1375 | 1122 | 254 | 137 (207) | 0.10 (0.15) | < 0.001 * (0.08, 0.12) |
| 142 | 119 | 95 | 25 | 10 (20) | 0.09 (0.16) | 0.02 * (0.05, 0.15) |
| 144 | 975 | 945 | 31 | 14 (30) | 0.01 (0.03) | < 0.01 * (0.01, 0.03) |
| Sum | 11277 | 9892 | 1386 | 770 (1216) | 0.07 (0.11) | < 0.001 * (0.06, 0.07) |

^a^ We present the frequencies for prey–predator and not predator–prey in the main results, because the frequencies of predator–prey sequences are the prey–predator frequency ± 1 value within each wildlife passage by year.

**Table S4.** Information on passage type and mammal sequence data **for 2012 only** (when populations were at their peak). See above (Table S1) for further details.

| **Passage** | **Number of sequences** | **Prey** | **Predators** | **Prey–predator sequences (*k_a_* (*k_e_*)** | **Proportions (*p_a_* (*p_e_*))** | ***P* (95 % CI)** |
| --- | --- | --- | --- | --- | --- | --- |
| 80 | 50 | 48 | 3 | 3 (3) | 0.06 (0.06) | 0.76 (0.01, 0.17) |
| 81 | 524 | 502 | 23 | 22 (22) | 0.04 (0.04) | 0.91 (0.03, 0.06) |
| 83 | 183 | 183 | 1 | 1 (1) | 0.01 (0.01) | 0.63 (0.00, 0.03) |
| 84 | 566 | 536 | 31 | 25 (29) | 0.04 (0.05) | 0.51 (0.03, 0.07) |
| 89 | 395 | 359 | 37 | 27 (33) | 0.07 (0.08) | 0.28 (0.05, 0.10) |
| 89.5 | 131 | 122 | 10 | 9 (10) | 0.07 (0.07) | 1.00 (0.03, 0.13) |
| 96 | 158 | 140 | 19 | 12 (17) | 0.08 (0.11) | 0.30 (0.04, 0.13) |
| 99 | 374 | 349 | 26 | 14 (24) | 0.04 (0.06) | 0.03 * (0.02, 0.06) |
| 104 | 12 | 4 | 9 | 3 (3) | 0.25 (0.21) | 0.73 (0.05, 0.57) |
| 107 | 1169 | 1156 | 14 | 13 (14) | 0.01 (0.01) | 1.00 (0.01, 0.02) |
| 110 | 26 | 19 | 8 | 5 (6) | 0.19 (0.21) | 1.00 (0.07, 0.39) |
| 122 | 74 | 50 | 25 | 14 (17) | 0.19 (0.22) | 0.58 (0.11, 0.30) |
| 124 | 1106 | 1038 | 69 | 57 (65) | 0.05 (0.06) | 0.37 (0.04, 0.07) |
| 125 | 537 | 511 | 27 | 23 (26) | 0.04 (0.05) | 0.69 (0.03, 0.06) |
| 133 | 784 | 676 | 109 | 84 (94) | 0.11 (0.12) | 0.31 (0.09, 0.13) |
| 142 | 51 | 35 | 17 | 8 (11) | 0.17 (0.22) | 0.31 (0.07, 0.29) |
| 144 | 535 | 524 | 12 | 8 (12) | 0.01 (0.02) | 0.37 (0.01, 0.03) |
| Sum | 6691 | 6252 | 440 | 328 (411) | 0.05 (0.06) | < 0.001 * (0.04, 0.05) |

**Table S5.** Information on passage type and mammal sequence data **for 2015 only** (when populations were crashing). See above (Table S1) for further details.

| **Passage** | **Number of sequences** | **Prey** | **Predators** | **Prey–predator sequences (*k_a_* (*k_e_*)** | **Proportions (*p_a_* (*p_e_*))** | ***P* (95 % CI)** |
| --- | --- | --- | --- | --- | --- | --- |
| 81 | 277 | 262 | 16 | 12 (15) | 0.04 (0.05) | 0.51 (0.02, 0.07) |
| 83 | 22 | 21 | 2 | 2 (2) | 0.09 (0.08) | 0.69 (0.01, 0.29) |
| 84 | 33 | 23 | 11 | 5 (7) | 0.15 (0.22) | 0.41 (0.05, 0.32) |
| 89 | 124 | 115 | 10 | 6 (9) | 0.05 (0.07) | 0.39 (0.02, 0.10) |
| 96 | 28 | 17 | 12 | 3 (7) | 0.11 (0.24) | 0.12 (0.02, 0.28) |
| 99 | 11 | 9 | 3 | 2 (2) | 0.18 (0.19) | 1.00 (0.02, 0.52) |
| 104 | 3 | 1 | 3 | 1 (1) | 0.33 (0.19) | 0.46 (0.01, 0.91) |
| 107 | 273 | 185 | 89 | 44 (60) | 0.16 (0.22) | 0.02 * (0.12, 0.21) |
| 110 | 4 | 3 | 2 | 1 (1) | 0.25 (0.24) | 1.00 (0.01, 0.81) |
| 122 | 44 | 35 | 10 | 6 (8) | 0.14 (0.17) | 0.69 (0.05, 0.27) |
| 124 | 129 | 103 | 27 | 20 (21) | 0.16 (0.16) | 0.91 (0.10, 0.23) |
| 125 | 74 | 55 | 20 | 14 (14) | 0.19 (0.20) | 1.00 (0.10, 0.30) |
| 133 | 77 | 66 | 12 | 7 (10) | 0.09 (0.13) | 0.40 (0.04, 0.18) |
| 142 | 8 | 9 | 0 | 0 (0) | 0.00 (0.00) | 1.00 (0.00, 0.37) |
| 144 | 173 | 174 | 0 | 0 (0) | 0.00 (0.00) | 1.00 (0.00, 0.02) |
| Sum | 1294 | 1078 | 217 | 123 (180) | 0.10 (0.14) | < 0.001 * (0.08, 0.11) |

**Table S6.** Information on passage type and mammal sequence data **for August only** (when juveniles are dispersing) from 2012 to 2015. See above (Table S1) for further details.

| **Passage** | **Number of sequences** | **Prey** | **Predators** | **Prey–predator sequences (*k_a_* (*k_e_*)** | **Proportions (*p_a_* (*p_e_*))** | ***P* (95 % CI)** |
| --- | --- | --- | --- | --- | --- | --- |
| 80 | 8 | 9 | 0 | 0 (0) | 0.00 (0.00) | 1.00 (0.00, 0.37) |
| 81 | 359 | 341 | 19 | 17 (18) | 0.05 (0.05) | 1.00 (0.03, 0.07) |
| 83 | 43 | 42 | 2 | 2 (2) | 0.05 (0.04) | 0.71 (0.01, 0.16) |
| 84 | 207 | 195 | 13 | 10 (12) | 0.05 (0.06) | 0.66 (0.02, 0.09) |
| 89 | 195 | 184 | 12 | 9 (11) | 0.05 (0.06) | 0.64 (0.02, 0.09) |
| 89.5 | 43 | 41 | 3 | 2 (3) | 0.05 (0.06) | 1.00 (0.01, 0.16) |
| 96 | 65 | 47 | 19 | 9 (13) | 0.14 (0.21) | 0.22 (0.07, 0.25) |
| 99 | 138 | 132 | 7 | 6 (7) | 0.04 (0.05) | 1.00 (0.02, 0.09) |
| 104 | 21 | 5 | 17 | 4 (4) | 0.19 (0.18) | 0.78 (0.05, 0.42) |
| 107 | 605 | 514 | 92 | 40 (78) | 0.07 (0.13) | < 0.001 * (0.05, 0.09) |
| 110 | 5 | 3 | 3 | 1 (1) | 0.20 (0.25) | 1.00 (0.01, 0.72) |
| 122 | 48 | 27 | 22 | 15 (12) | 0.31 (0.25) | 0.32 (0.19, 0.46) |
| 124 | 426 | 373 | 54 | 32 (47) | 0.08 (0.11) | 0.02 * (0.05, 0.10) |
| 125 | 195 | 161 | 35 | 25 (29) | 0.13 (0.15) | 0.54 (0.08, 0.18) |
| 133 | 294 | 204 | 91 | 53 (63) | 0.18 (0.21) | 0.18 (0.14, 0.23) |
| 142 | 17 | 15 | 43 | 3 (2) | 0.18 (0.14) | 0.72 (0.04, 0.43) |
| 144 | 228 | 225 | 4 | 4 (4) | 0.02 (0.02) | 0.80 (0.00, 0.04) |
| Sum | 2913 | 2518 | 396 | 232 (342) | 0.08 (0.12) | < 0.001 * (0.07, 0.09) |

**Figure S1.** Number of observations of prey–prey (0-0), prey–predator (0-1), predator–prey (1-0), and predator–predator (1-1) sequences in 17 wildlife passages monitored from 2012 to 2015.

**Figure S2.** Non-zero observed proportion of a prey–predator sequence with 95 % confidence intervals (*p_a_*) obtained from binomial tests compared to the corresponding expected (null) probabilities of a prey–predator sequence (*p_e_*) for when the prey-trap was expected to be most likely to occur. (**a**) population peak in 2012; (**b**) population crash in 2015; and (**c**) when juveniles are most prevalent and dispersing (August) for 2012 to 2015.

**Figure S3.** Observed proportion of a prey–predator sequence (*p_a_*) with 95 % confidence intervals (*p_a_*) obtained from binomial tests compared to the corresponding expected (null) probabilities of a prey–predator sequence (*p_e_*) based on (**a**) wildlife passage type (predators have higher use of specific wildlife passage types over others; DCC: *n* = 7, DWC: *n* = 4, and PC: *n* = 6); and (**b**) passage openness (a proxy for visibility, calculated as width × height/length ^31^, closed: *n* = 11 and open: *n* = 6).
